# The Diagnosis of Scarlet Fever

**Published:** 1905-02-11

**Authors:** 


### THE DIAGNOSIS OF SCARLET FEVER.

The man who shall provide a pathognomonic index for scarlet fever will deserve well of the profession, for, so far, extension and amplification of knowledge in the matter have but darkened counsel. This is not to disparage those who have attacked the subject; the task is a worthy one, but up to the present its prosecution has only emphasised the recognised difficulties and introduced obstacles not generally suspected. Such will probably be the verdict upon the statements of Dr. Poynton and Dr. J. D. Rolleston in their recent communications.[1]

Briefly stated, the position is as follows: The disease with which scarlet fever is most likely to be confounded is german measles, which, though usually affecting a morbilliform type of exanthem, not infrequently appears in the form of a generalised erythema. If we admit the existence of the "fourth disease" of Dr. Clement Dukes, a third alternative awaits the diagnostician. These three, then, are all marked by mild constitutional symptoms (for it is only the mild cases of scarlet fever which admit of doubt) — a scarlatiniform eruption and subsequent gross desquamation. There is yet one more item in the catalogue of doubts—viz., the prodromal fleeting scarlatiniform rash which occasionally heralds variola, and, according to Rolleston, both varicella and measles. These we may dismiss without much comment, for in their case the doubt in diagnosis is spontaneously resolved. It may be noted, however, that in the case of measles the prodromal rash occurs most often on the first day of the illness and may consist of a variety of mixed erythemata concurring in the same patient. It is clearly most important to recognise the occurrence of these prodromal rashes, and the moral of them is the avoidance of precipitancy in making a diagnosis.

For the others, it is not too much to say that they cannot, on occasions, be distinguished. In every epidemic of undoubted scarlet fever there occur atypical cases lacking one element or more of the orthodox disease. Thus, one case in a series may have sore throat and fever without a rash, and yet desquamate; another may desquamate without any observed illness to account for the skin condition. Similarly, where many children are collected, as in the wards of a children's hospital, there undoubtedly occur exanthemata which breed true, and yet are neither orthodox scarlet fever nor orthodox German measles, though the rash is scarlatinal. We have ourselves witnessed such an epidemic. The incubation period was about 12 days, the rash—a diffuse erythema, lacking the typical punctate character of scarlet fever—appeared within 24 hours, the throat was injected, and the temperature rose to about 102° F. on the first night. There was no glandular enlargement, the fever fell by the end of the second day as a rule, the desquamation was copious, and none of the patients developed albuminuria, nor did any cases of typical scarlet fever mark the series.

It is possible that this was the "fourth disease," but in all cases of this class merely clinical characteristics afford no certain clue. Until the morbific elements of the alternative diseases are identified, diagnosis must often remain a mere matter of opinion, and it is better in a doubtful case to assume a certainty not scientifically justifiable and treat it as scarlet fever, than to have an optimistic doubt disconcerted by the appearance of grave nephritis, or of other serious complication which establishes the scarlatinal character of the infection.

[1] Brit. Med. Jour., Feb. 4, 1905.

